# UDP-glycosyltransferases alleviate the toxic effects of deoxynivalenol on the growth performance and gut damage of Kunming mice

**DOI:** 10.1038/s41598-025-02712-6

**Published:** 2025-05-23

**Authors:** Jiaxu Liu, Xue Ling, Zhaoquan Chen, Huajie Yang, Sitao Guo, Bingyang Zhou, Pengwei Zhu, Zheng Yang, Yongqiang Wang

**Affiliations:** Key Laboratory of Microecological Resources and Utilization in Breeding Industry, Ministry of Agriculture and Rural Affairs, Guangdong HAID Group Co., Ltd, Guangzhou, China

**Keywords:** Deoxynivalenol, UDP-glucosyltransferase (UGT), Mice, Detoxification, Toxicology, Clinical pharmacology

## Abstract

The purpose of this study was to assess the effects of UDP-glycosyltransferases (UGTs) on alleviating the toxic effects of deoxynivalenol (DON) on Kunming mouse growth performance and gut damage. In this study, a total of 60 3-week-old male Kunming mice were randomly divided into 4 groups and fed the following dietary and drug treatments for 7 weeks: CON, basal diet; CTX, basal diet with i.p. injection of cyclophosphamide (CTX); CTX + DON, basal diet with 12 mg/kg DON and i.p. injection of cyclophosphamide; and CTX + DON + UGTs, basal diet with 12 mg/kg DON and UGTs 1 mg/kg and i.p. injection of cyclophosphamide. Compared with those in the CON group, the growth performance, serum immunoglobulin contents (IgG), antioxidant defense enzyme activities(SOD), intestinal barrier integrity and permeability (the ratio of villi length to crypt depth), tight junction proteins (occludin and claudin 5) expression, intestinal cell apoptosis (Bcl-2), and histopathological lesions in the guts of the DON- and CTX-treated mice were significantly lower (*p* < 0.05). These negative effects on DON-exposed mice were significantly mitigated when the mice received a UGT-supplemented diet (1 mg/kg) (*p* < 0.05). We concluded that UGTs could serve as dietary supplements to treat intestinal disorders associated with DON-induced growth-retardation in animals.

**Supplementary Information:**

The online version contains supplementary material available at 10.1038/s41598-025-02712-6.

## Introduction

Mycotoxin is a secondary metabolite produced by fungi, and many studies have shown that mycotoxins pose significant risks to human and animal health^1^. The Food and Agriculture Organization of the United Nations (FAO) estimated that 25% of crop production worldwide is contaminated with mycotoxins yearly^2^. According to the China Grain Development Report of 2019, the detection rate of mycotoxins in grains is greater than 90% in China, and 21 million tons of grain and cereal are contaminated with mycotoxins every year, with direct or indirect economic losses reaching 24 billion yuan and more than 100 billion yuan, respectively^3^. Deoxynivalenol (DON), or vomitoxin, is one of the major mycotoxins that causes grain pollution. A recent review revealed that the proportions of DON-positive samples in East Asia, Northern Europe, Central Europe, Central America, North America, and South Africa were 84.8%, 74.2%, 69.8%, 70.0%, 64.1%, and 63.2%, respectively^4^.

DON is produced by mainly *Fusarium graminearum*, which is a type-B trichothecene mycotoxin^5^. The main toxic effects of DON are cytotoxicity, immunotoxicity, neurotoxicity, and synergistic toxicity with other biological toxins. The ingestion of feed or food contaminated with DON can have deleterious effects on humans and animals, such as anorexia, vomiting, diarrhea, and abdominal pain. Long-term exposure to DON can result in immunosuppression, nutritional deficiencies, growth retardation, and dysplasia of reproductive organs^6^. Additionally, the intestine is the primary target organ through which DON exerts toxic effects, accompanied by destruction of the normal physiological function of the intestine^7–9^. Intact intestinal barrier function is essential for maintaining the normal growth and production of animals.

At present, increasing evidence illustrates that adding mycotoxins detoxifying enzymes to feed is an effective strategy to alleviate the damage caused by mycotoxins to the animal body^10,11^. UDP-glycosyltransferases (UGTs) are the most widely studied enzymes that confer DON resistance via the conjugation of DON to DON-3-O-glucoside (D3G)^12^. The first identified UGT that detoxified DON was DOGT1. DOGT1 catalyzes the transfer of glucose to DON, resulting in the formation of the less toxic D3G and increased DON tolerance in *Arabidopsis* via the overexpression of this gene^13^. Moreover, many UGT genes from plants, such as *HvUGT-10W1*^14^, *TaUGT12887*/*TraesCS5B02G148300*^15^, *HvUGT13248*^16^, *TaUGT3* and *TaUGT5*^17,18^, have been shown to detoxify DON. UGTs, the most important enzymes involved in phase II metabolism, are a multigenic and highly divergent superfamily of enzymes that are widely found in all living organisms, many of which are related to disease resistance^19^. DON glycosylation by UGTs appears to be the principal detoxification mechanism of DON^20,21^. As a major phase II drug metabolizing enzymes in mammals, UGTs not only control the concentration of endogenous compounds to maintain normal homeostasis, but also bind exogenous substrates such as drugs and plant secondary metabolites to avoid exogenous toxic effects^22–24^. In light of these functions, UGTs possess latent value as additives in animal feed.

However, few reports on the applications of the UGT cloned from *Bos taurus* and expressed by *Saccharomyces cerevisiae* exist, and its shielding effect and potential mechanism for DON-induced intestinal epithelial injury remain unclear. Cyclophosphamide (CTX) is a major constituent of cancer chemotherapy agent and widely used in the treatment of various types of cancer^25^, but CTX treatment can lead to immunosuppression. Therefore, many immunosuppressed mice models were established using CTX^26,27^. In our study, when mice were given feed contaminated with DON at a concentration of 12 mg/kg, and i.p. injection of cyclophosphamide, gut injury model could be established effectively. This study aimed to determine the protective effect of UGTs on the intestinal epithelial barrier of DON-exposed mice and elucidate the underlying mechanism, providing reference information for the use of UGTs.

## Results

### Growth performance

**Fig. 1:**
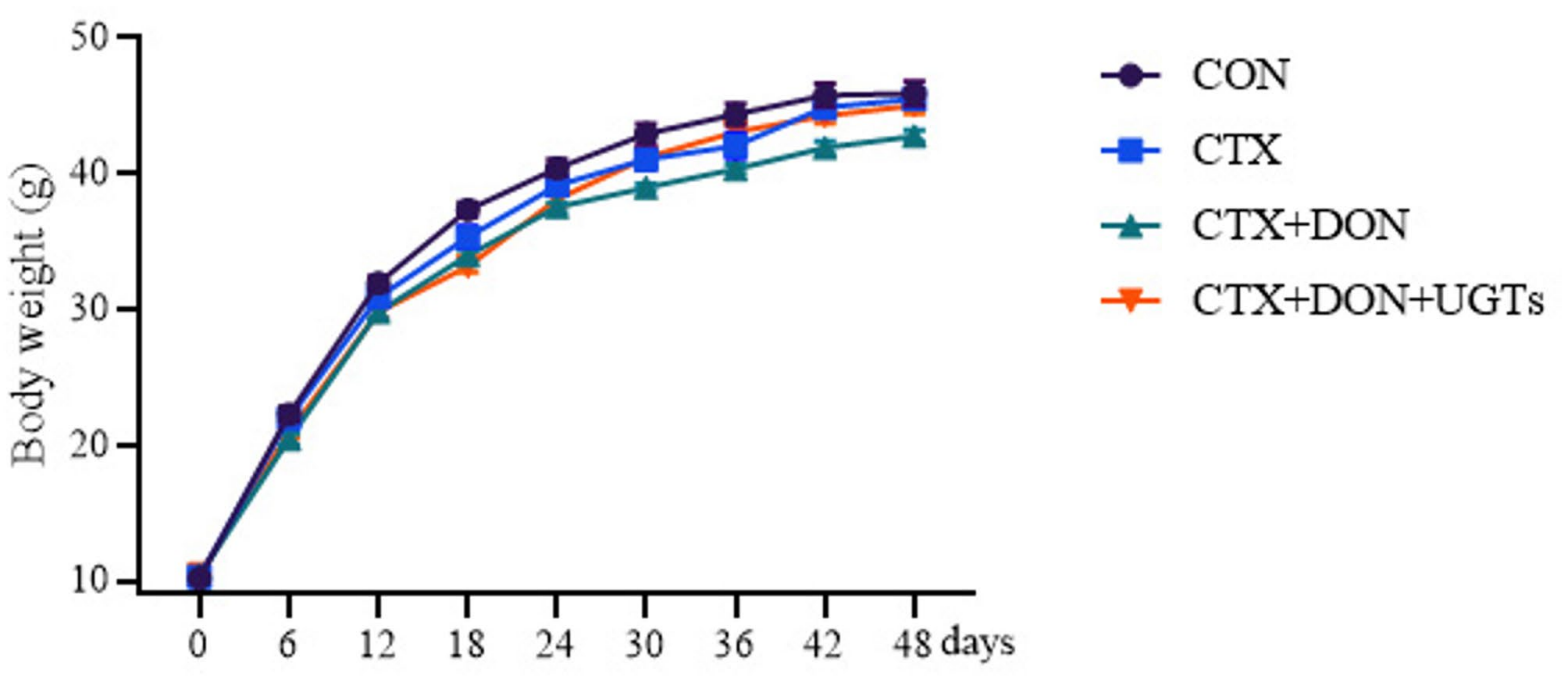
Weight changes.

Weight gain trends over time of mice are shown in Fig. 1, there are similar weight gain trends among groups. The effects of the dietary treatments on growth performance are shown in Table 1. The lowest final weight (FW) was observed in the CTX + DON group (*p <* 0.05) compared with the other groups. After the model mice received the UGTs, a significant improvement in growth performance in terms of FW was observed (*p <* 0.05).

**Table 1:** Growth performance of Kunming mice in each group.

Parameters	Groups			
	CON	CTX	CTX + DON	CTX + DON + UGTs
IW (g)	10.22 ± 0.28^a^	10.32 ± 0.36^a^	10.41 ± 0.29^a^	10.48 ± 0.37^a^
FW (g)	45.90 ± 0.88^a^	45.52 ± 0.74^a^	42.77 ± 0.45^b^	45.03 ± 0.70^a^
ADG (g/day)	0.64 ± 0.16^a^	0.64 ± 0.14^a^	0.61 ± 0.14^a^	0.62 ± 0.13^a^
ADFI (g/day)	5.63 ± 0.29^a^	5.79 ± 0.31^a^	5.66 ± 0.33^a^	5.64 ± 0.30^a^
FCR (feed/gain)	8.80 ± 2.09^a^	9.44 ± 2.02^a^	7.42 ± 1.55^a^	7.37 ± 2.00^a^

IW, initial weight; FW, final weight; ADFI, average daily feed intake; ADG, average daily gain; FCR, feed conversion ratio.

Within the same indicator, different letters indicate significant differences (*p* < 0.05), whereas the same letter indicates insignificant differences (*p* > 0.05).

### Histopathology

**Fig. 2:**
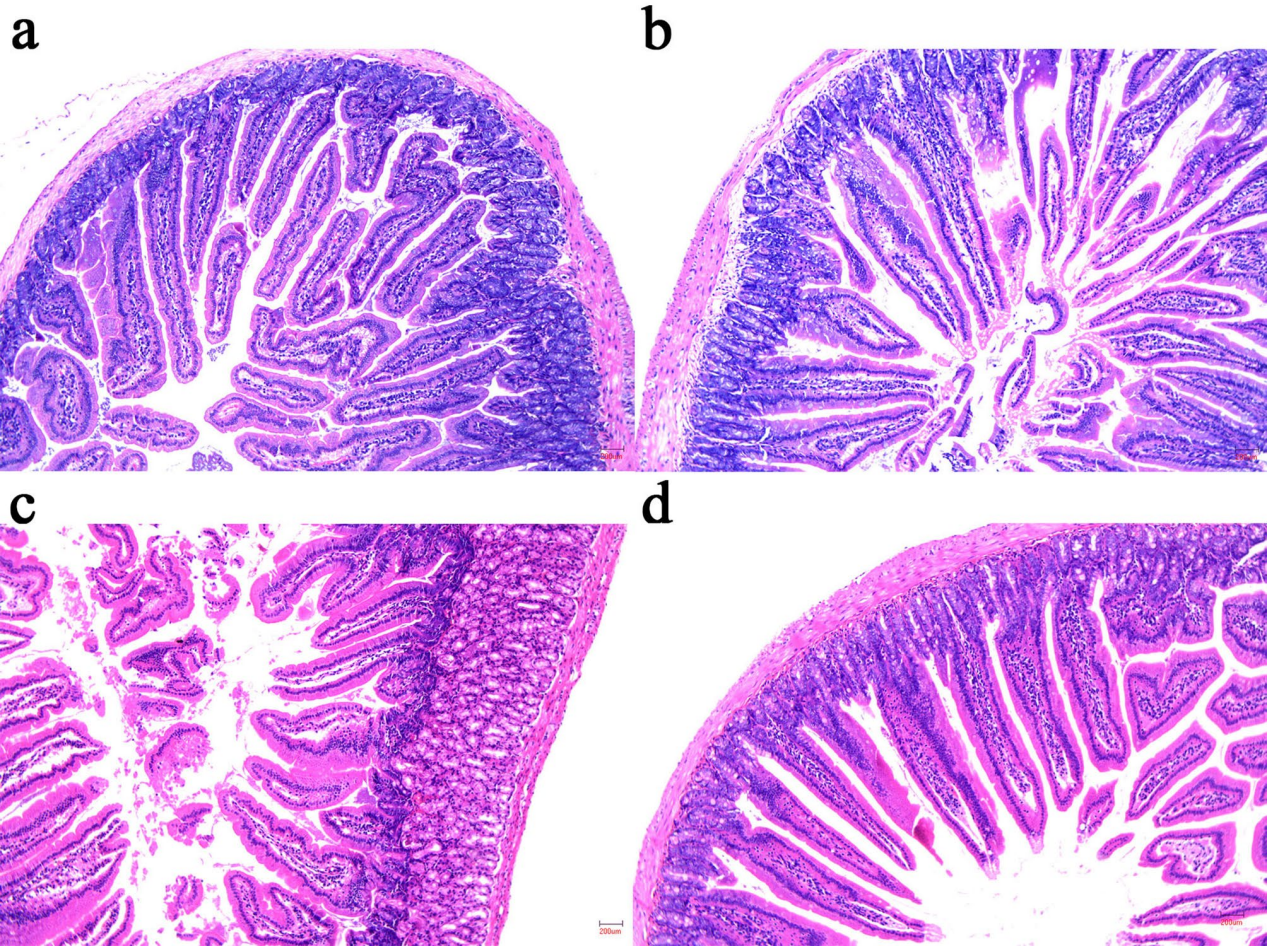
Results of duodenal pathological observation (×100). (**a**) CON; (**b**) CTX; (**c**) CTX + DON; (**d**) CTX + DON + UGTs.

**Fig. 3:**
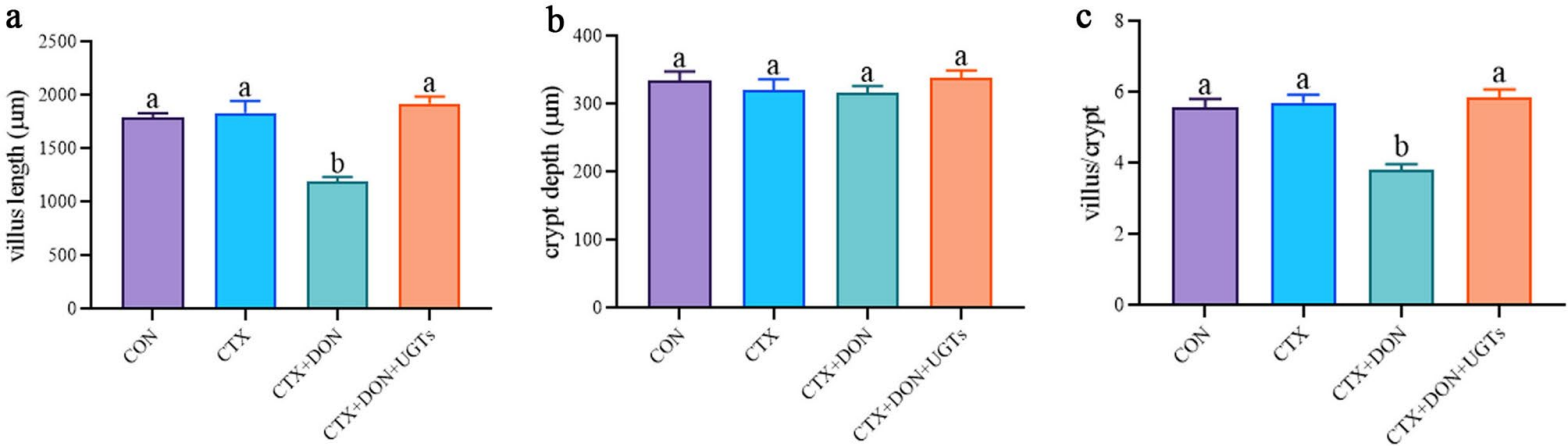
(**a**) Villus length, (**b**) crypt depth, and (**c**) villus length/crypt depth (*n* = 3). The groups with different letters are significantly different (*p* < 0.05).

Figure 2 shows the pathological results for the duodenum in each group. The morphological results revealed that the muscle layer, lamina propria, and mucosal layer of the duodenum in the CON group and the CTX group remained intact, and no necrosis or shedding of the intestinal epithelium was observed. The intestinal villi of the CTX + DON group mice exhibited breakage, shortening, severe atrophy, and disordered arrangement. In contrast, the CTX + DON + UGTs group presented well-delineated villi, and atrophy or apical necrosis of the villi was not observed. Similarly, both the value of the villus length and the ratio of villus length to crypt depth were the lowest in the CTX + DON group. In contrast, in the CTX + DON + UGTs group, an increased villous height and a greater ratio of villi to crypts in the duodenum were observed (*p* < 0.05) (Fig. 3). Importantly, after DON-exposed and CTX-treated mice received UGT treatment, the intestinal pathological changes were significantly reversed.

### Serum D-lactate levels

**Fig. 4:**
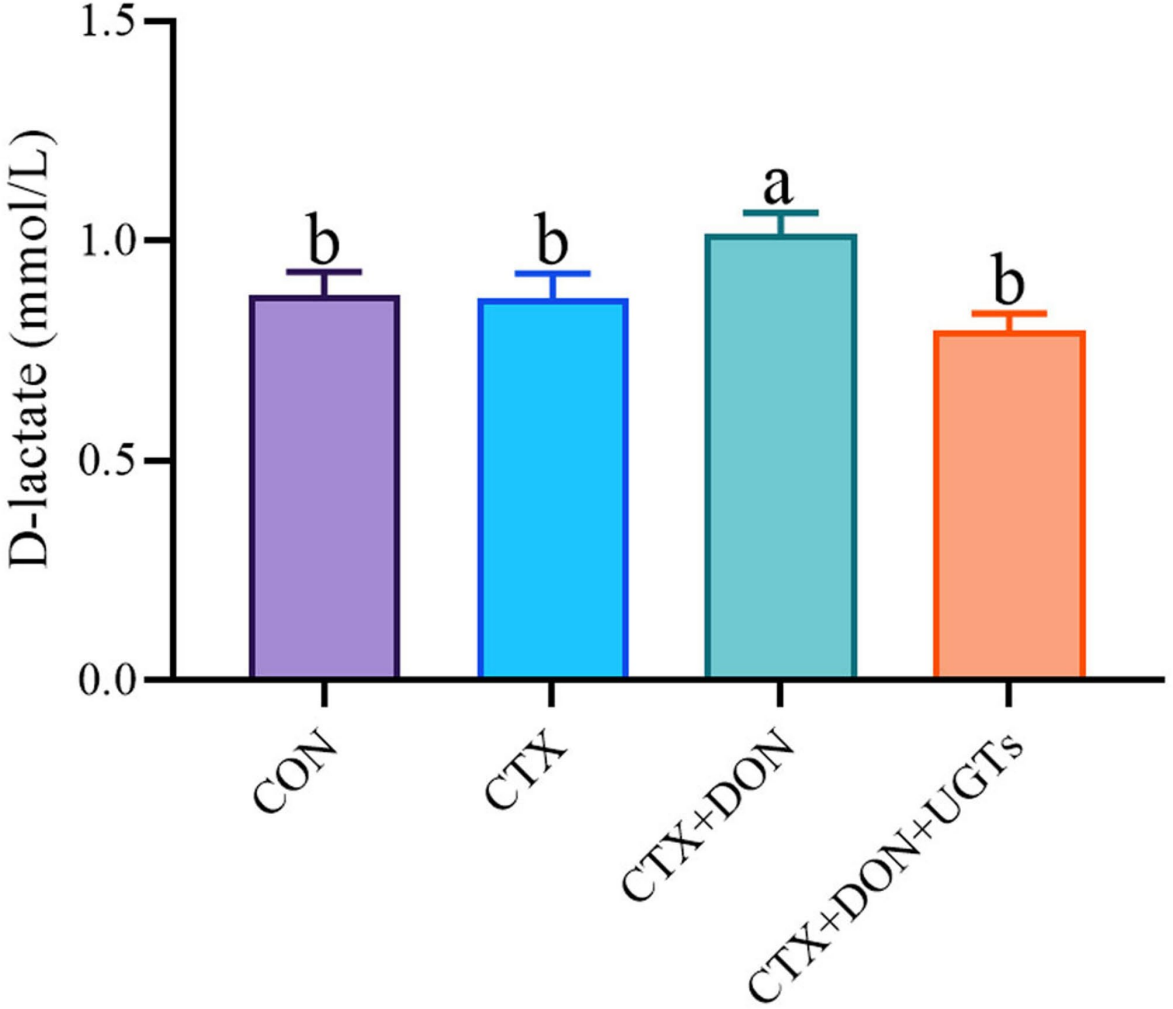
D-lactic acid levels in the serum of mice. The groups with different letters are significantly different (*p* < 0.05).

Serum D-lactate levels are positively correlated with the degree of intestinal permeability^28^, and the results are shown in Fig. 4. Serum D-lactate levels in CTX + DON group mice were significantly increased compared with the other groups (*p* < 0.05). The experimental results indicate that UGTs could significantly reduce the intestinal permeability of DON-exposed and CTX-treated mice.

### Oxidative stress assay using liver samples

**Fig. 5:**
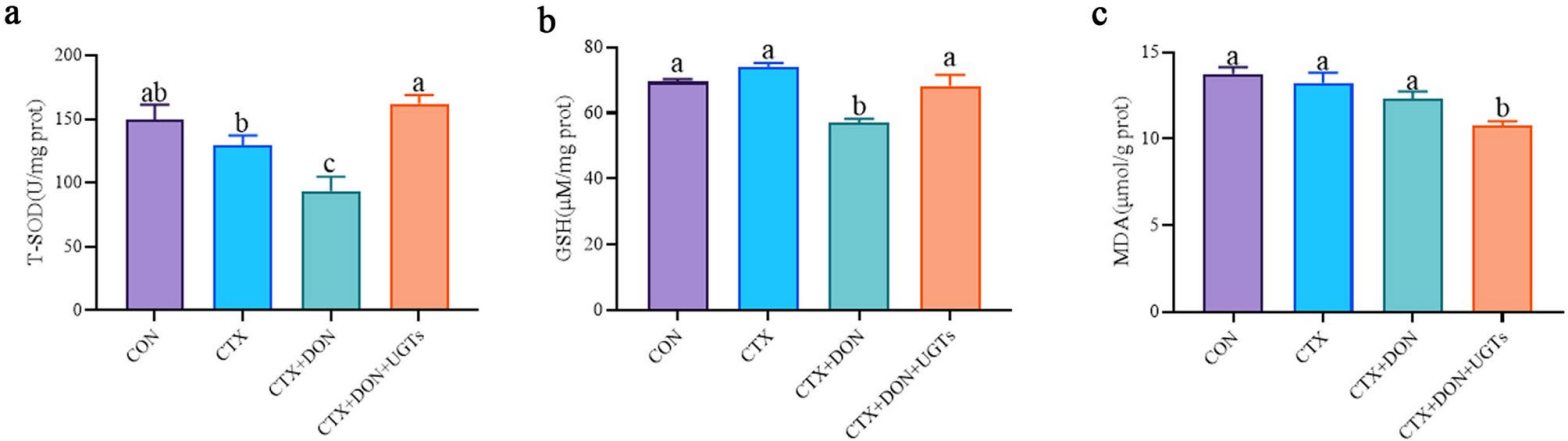
Experimental results of oxidative stress in the liver. (**a**) T-SOD, total superoxide dismutase; (**b**) GSH, glutathione; (**c**) MDA, malonic dialdehyde. The groups with different letters are significantly different (*p* < 0.05).

The total superoxide dismutase (T-SOD) activity and the contents of glutathione (GSH) and malonic dialdehyde (MDA) in the livers of the mice were detected, and the results are shown in Fig. 5. Compared with those in the CON group, the T-SOD activity and GSH content in the livers of the mice in the CTX + DON group were significantly lower (*p* < 0.05). After intervention with UGTs, the SOD activity and GSH content in the livers of DON-exposed and CTX-treated mice significantly increased (*p* < 0.05), and MDA levels significantly decreased (*p* < 0.05).

### Serum IgG levels

**Fig. 6:**
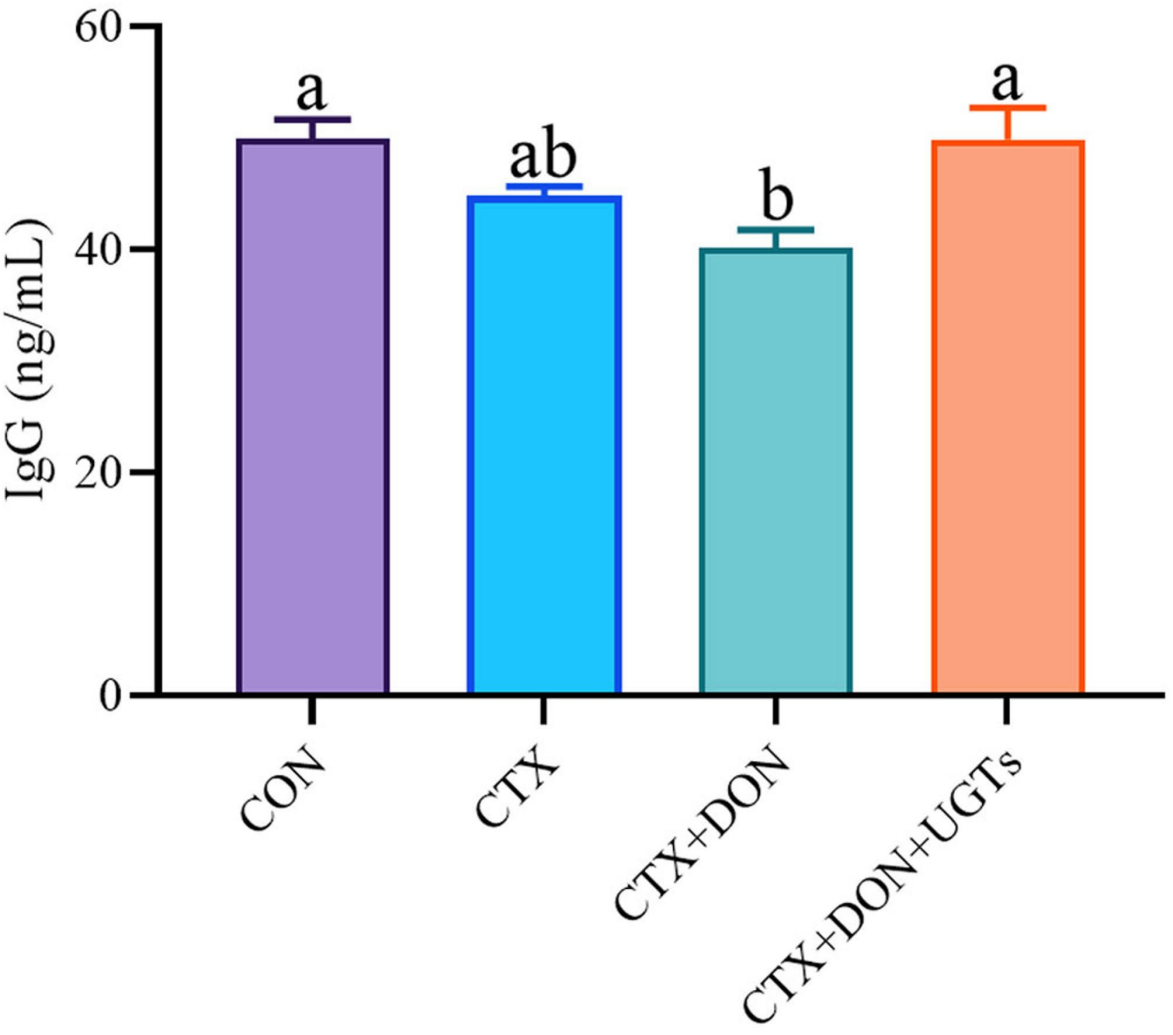
Serum IgG levels in mice. The groups with different letters are significantly different (*p* < 0.05).

As shown in Fig. 6, serum immunoglobulin G (IgG) levels were assessed in all mice. Compared with that in the CON group, the serum IgG content in the CTX + DON group was significantly lower (*p* < 0.05). Serum IgG levels in DON-exposed and CTX-treated mice fed UGTs were significantly increased (*p* < 0.05).

### Tight junction protein-related gene expression analysis using qRT‒PCR

**Fig. 7:**
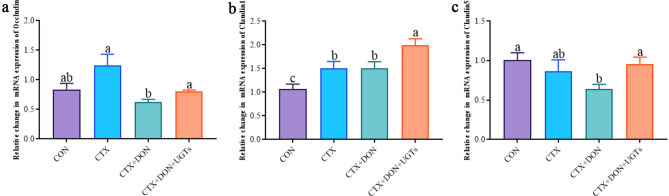
Relative expression of tight junction protein genes in the duodenum. (**a**) Occludin, (**b**) claudin 1, and (**c**) claudin 5. The groups with different letters are significantly different (*p* < 0.05).

As shown in Fig. 7, the relative expression level of the occludin gene in the CTX + DON group was significantly lower than that in the CTX group (*p* < 0.05). Compared with that in the CON group, the relative gene expression level of Claudin 5 in the CTX + DON group was also significantly lower (*p* < 0.05). Compared with those in the CTX + DON group, the relative expression levels of the occludin, claudin 1 and claudin 5 genes in the CTX + DON + UGTs group were significantly greater (*p* < 0.05). The experimental results revealed that UGTs may exert a protective effect on the intestinal barrier by regulating the expression of tight junction proteins.

### Inflammation- and cell apoptosis-related gene expression analysis using qRT‒PCR

**Fig. 8:**
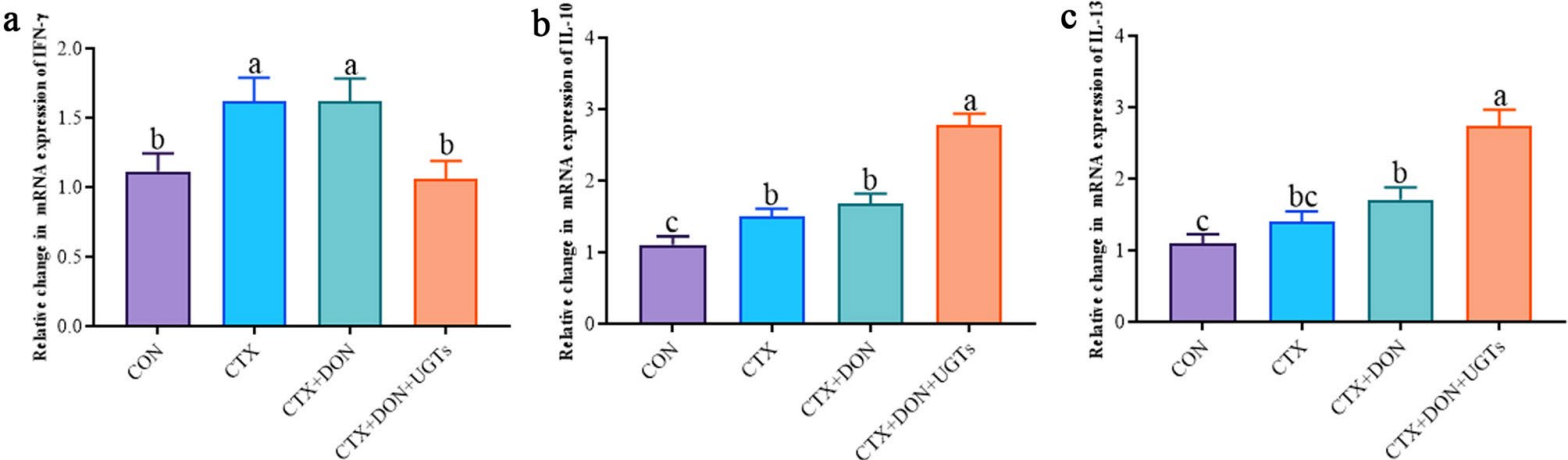
Relative expression of inflammatory genes in the duodenum. (**a**) IFN-γ, (**b**) IL-10, and (**c**) IL-13. The groups with different letters are significantly different (*p* < 0.05).

As shown in Fig. 8, compared with those in the CTX + DON group, the relative expression level of the IFN-γ gene was significantly lower (*p* < 0.05), and the relative expression levels of the IL-10 and IL-13 genes were significantly greater in the CTX + DON + UGTs group (*p* < 0.05). These results indicate that UGTs can regulate abnormal cell apoptosis and alleviate intestinal inflammation in mice treated with DON and CTX.

**Fig. 9:**
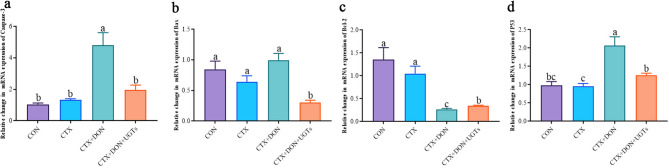
Relative expression of apoptosis factor genes in the duodenum. (**a**) Caspase-3, (**b**) Bax, (**c**) Bcl-2, and (**d**) P53. The groups with different letters are significantly different (*p* < 0.05).

After DON exposure, intestinal epithelial cells are attacked first by high concentrations of vomitoxins, leading to abnormal cell apoptosis and an inflammatory response, as shown in Fig. 9. Compared with those in the CON group, the relative expression levels of the Caspase-3 and P53 genes in the CTX + DON group were significantly greater (*p* < 0.05), whereas the relative expression level of the Bcl-2 gene in the CTX + DON group was significantly lower (*p* < 0.05). After UGT intervention, the relative expression levels of the Caspase-3 and P53 genes in DON-exposed model mice returned to normal levels, and the relative Bax expression level was significantly reduced. Moreover, compared with that in the CTX + DON group, the relative expression level of the Bcl-2 gene in the CTX + DON + UGTs group was significantly greater (*p* < 0.05).

## Discussion

In our preliminary study, 10 mg/kg DON did not lead to obvious growth inhibition or intestinal damage in weanling mice after 60 days. Therefore, we used cyclophosphamide (CTX) as an immunosuppressant and more DON (12 mg/kg) to establish a DON-mediated mouse model of intestinal damage^29^. To observe whether CTX affects the experimental results, we established a CTX group. Comparing the CON group with the CTX group, we detected that CTX had no effect on growth performance or intestinal health.

DON pollution is widespread in cereals and poses a serious threat to human and animal health. In this study, we observed that the final weight (FW) of the mice in the CTX + DON group was lower than that in any other group, and no significant difference in FW was noted between the CTX + DON + UGTs group and the CON group. The results showed that oral UGTs could increase the final weight and improve the worsening of animal conditions caused by DON.

When animals are exposed to DON via the oral route, six negative effects on the body are observed: (1) cytotoxicity; (2) steroidogenesis disruption; (3) intestinal barrier disruption and increased intestinal permeability; (4) oxidative stress; (5) inhibition of cellular protein synthesis; and (6) ribosomal stress syndrome, such as cell apoptosis^30,31^. In this study, a model was established in Kunming mice with reduced growth performance and intestinal damage, and UGTs alleviated the effects of DON in these mice by increasing intestinal permeability and intestinal tissue damage. Mammals express 4 families of UGTs which use mainly UDP-glucuronic acid as sugar donor, and has detoxification and homeostasis function^32^.

### The phenotype of increased intestinal permeability

The gastrointestinal tract of animals is the primary target organ of toxins. DON can cause serious histopathological changes in the intestines of many animals, including rodents, pigs, and chickens^33–35^. The length of the villi in the small intestine, the depth of the crypt, and their ratio are often used as important indicators of intestinal villi integrity^33^. In this study, obvious histopathological changes in the mice treated with DON and CTX were observed, including rupture of the intestinal villi, necrosis of the mucosal epithelium, and shortening of the length of the small intestinal villi. In addition, no significant differences were noted between the CTX + DON + UGTs group and the CON group, indicating that UGTs could protect intestinal barrier integrity.

Intestinal health depends on the physical barrier, chemical barrier, immune barrier, and biological barrier of the intestinal tract. Of these, the physical barrier is the basis of intestinal integrity. The implementation of physical barrier function is inseparable from the sealing effect of tight junction proteins on intercellular gaps^36,37^. The decreased expression of tight junction proteins is accompanied by an increase in intestinal permeability. Claudins are a family of proteins responsible for the formation of tight junctions between intestinal cells. Claudins can also interact with other tight junction proteins, such as zonula occludens-1 (ZO-1), via the PDZ motif containing the carboxyl end^38^. Claudins classified into the “pore-sealing” group and “pore-forming” group. The pore-sealing group, which can seal the pores between intestinal cells to reduce permeability, includes claudin-1, -3, -4, -5, -7 and − 19, and the pore-forming groups can form pores between intestinal cells, decreasing the tightness of the epithelium and increasing its permeability, including that of claudin-2 and claudin-15^39,40^. Pore-forming claudins are responsible for the size and charge selectivity of paracellular transport via tight junctions in a large variety of epithelia^41,42^. In this study, the relative expression of the Occludin and Claudin5 genes in the duodenum of the CTX + DON group was the lowest among the four groups. Although the relative expression of the Occludin, Claudin1 and Claudin5 genes in the duodenum of mice receiving UGTs significantly increased, we hypothesized that UGTs could repair the intestinal barrier by increasing the expression of Occludin and Claudins.

D-lactate is usually regarded as an important indicator of intestinal barrier damage and permeability changes^36,43^. In this study, serum D-lactate levels in CTX + DON group mice were significantly greater than those in the other three groups (*p* < 0.05), which was consistent with obvious pathological changes and decreased expression of the Occludin and Claudin5 genes. These experimental results indicate that UGTs can decrease intestinal permeability by maintaining the integrity of the intestinal barrier.

### The phenotype of increased intestinal cell injury

First, studies have shown that DON can cause an imbalance in the redox system in the liver^44,45^ and seriously interferes with the intestinal immune balance^8^. GSH, SOD, CAT, and GSH-Px are important components of the endogenous antioxidant defense system and play important roles in free radical scavenging and maintaining the intracellular redox balance^46^, and MDA is the main byproduct of lipid peroxidation^47^. Other studies have shown that, compared with those of the control group, the activities of SOD, CAT, and GSH-Px and the content of GSH decreased, and the level of MDA increased when porcine splenic lymphocytes were exposed to DON even at the lowest doses^48^. In the present study, in the CTX + DON group, SOD activity and GSH levels in the liver were significantly reduced (*p <* 0.05). After the model mouse received the diet contaminated with UGTs, those negative impacts were eliminated. Interestingly, MDA levels in the mice in the CTX + DON + UGTs group were lower compared with the other groups. On the basis of these experimental results, we hypothesize that UGTs alleviate the cellular damage caused by oxidative stress.

Second, the intestinal tract is not only a digestive organ but also the largest immune organ^49,50^. Many studies have shown that DON strongly interferes with the intestinal immune balance^51^, which is closely related to intestinal barrier function. IFN-γ is the main proinflammatory cytokine and can increase intestinal permeability^52^. IL-10 and IL-13 are important anti-inflammatory cytokines that bind to protect the intestinal barrier. Knockout of the IL-10 gene in mice results in increased intestinal permeability and intestinal inflammation^53,54^. The microflora promotes the production of IL-10 by intestinal macrophages and then regulates the integrity of the intestinal epithelium and maintains intestinal homeostasis^54–56^. IL-13 stimulates the production of trefoil factor 3 (TFF3) and restin-like molecule β (RELM β), which play important roles in epithelial repair, mucosal protection and mucin stabilization^57,58^. In this study, compared with those in the CON group, relative IFN-γ expression levels in the CTX + DON group and relative IL-10 and IL-13 expression levels in the CTX + DON + UGTs group were significantly greater (*p <* 0.05), indicating that inflammation had already occurred and that organization repair was ongoing.

Finally, DON leads to abnormal intestinal cell apoptosis by activating the MAPK signaling pathway^59^. The process of DON-induced apoptosis is related to Caspase-3 activation and the expression of several proteins in the Bcl-2 family^60,61^. Activated caspase-3 is the direct executor of apoptosis. Bax is a proapoptotic protein, and Bcl-2 is an antiapoptotic protein. P53 can upregulate Bax expression and downregulate Bcl-2 expression to promote apoptosis^62,63^. Abnormal cell apoptosis can result in intestinal barrier dysfunction and increased permeability. In this study, the relative expression levels of the Caspase-3 and P53 genes in the CTX + DON group were significantly increased (*p <* 0.05), whereas the relative expression level of the Bcl-2 gene in the CTX + DON group was significantly reduced (*p <* 0.05). Interestingly, in the CTX + DON + UGTs group, relative Caspase-3 and P53 expression levels returned to normal. The relative expression levels of Bax were the lowest compared with those in the other groups, and the relative expression levels of the Bcl-2 genes were significantly increased. These results indicate that UGTs alleviate intestinal epithelial injury and protect intestinal barrier function by regulating the expression of apoptotic factors.

Compared with those in the CON group, the growth performance of the intestinal villi in the DON and CTX groups was unsatisfactory, and the antioxidant defense enzyme activities were negatively altered. In addition, the expression of inflammatory cytokines and proapoptotic genes was increased, and the expression of apoptosis suppressor genes and tight junction protein genes was decreased. IgG synthesis was also inhibited in the DON + CTX group (*p* < 0.05). We found that UGTs could reverse the damage caused by DON. However, there are still some limitations in these experiments. Although it is already clear that UGTs can efficiently degrade DON in vitro, those are still unclear whether UGTs directly or indirectly affect the metabolism of DON in biological systems and UGT activity could be affected by gut microbiota composition, which requires further exploration in the future. As a potential commercial application of UGTs in animal feed additives, dietary supplementation with UGTs has protective effects against damage caused by DON in Kunming mice, but the activity of UGTs could be inhibited by natural polyphenol compound, such as piceatannol^64^ and carvacrol^65^. However, our experimental results clearly indicate that dietary supplementation with UGTs has protective effects against damage caused by DON in Kunming mice, providing a reference for the use of UGTs in animal husbandry.

## Conclusions

The results of the present study provide significant evidence of the potential protective effects of UGTs against DON-induced intestinal epithelium injuries in mice. UGTs effectively repaired DON-induced intestinal damage, as evidenced by improved intestinal structure and gut barrier functions. Moreover, UGTs attenuated oxidative stress and enhanced immunity in DON-challenged mice to improve their growth performance. This study revealed that UGTs could potentially serve as effective additives to protect against DON-induced hazardous effects in animals. In addition, UGT might be utilized as an ingredient in animal nutritional supplements or feed formulas in the future.

## Materials and methods

### Preparation of feed

The basal diet consisted of 1 L of water mixed with 2 kg of feed powder (Xietong Pharmaceutical Bioengineering, Jiangsu, China). Then, the mixture was made into a uniform shape and dried in an oven at 60 °C for 20 h.

Basal diet with 12 mg/kg DON: Briefly, 100 mL of DON (Guelph, Ontario, Canada) at a concentration of 240 mg/kg was diluted in 1 L of water and then mixed with 2 kg of feed powder (Xietong Pharmaceutical Bioengineering, Jiangsu, China). The mixture was made uniform and dried in an oven at 60 °C for 20 h.

Basal diet with 12 mg/kg DON and 1 mg/kg UGTs: Here, 100 mL of DON (Guelph, Ontario, Canada) at a concentration of 240 mg/kg and 2 mg of UGTs was diluted in 1 L of water and then mixed with 2 kg of feed powder (Xietong Pharmaceutical Bioengineering, Jiangsu, China). The mixture was made uniform and dried in an oven at 60 °C for 20 h.

### Experimental animals and treatments

All animal protocols described in this study were performed in accordance with the ARRIVE Guidelines. The experimental procedure was approved by the Institutional Animal Care and Use Committee of Jilin University (approval no. JLU-20150226) and were performed in strict compliance with the requirements of the Animal Ethics Procedures and Guidelines of the People’s Republic of China. Deoxynivalenol detoxifying enzymes (UGTs) are produced from *Saccharomyces cerevisiae* expressing UGT genes from *Bos taurus*.

On the basis of preliminary experiments, 3-week-old male Kunming mice were selected as experimental subjects, the dosages of DON, UGTs and cyclophosphamide (CTX) were confirmed, and cyclophosphamide was shown to play a role in facilitating the establishment of a model with damaged intestines^29^. These mice were purchased from the Experimental Animal Center of Southern Medical University and maintained in clean, pathogen-free animal cages with unrestricted access to standard laboratory food and water on a 12-hour light/dark cycle and in an environment with regulated humidity (60-80%) and temperature (22 ± 1˚C). Sixty male Kunming mice were randomly divided into four groups (*n* = 15): the CON group, CTX group, CTX + DON group, and CTX + DON + UGTs group. The experiment lasted for 48 days, and the treatment methods are shown in Table 2. The weight and food intake of each group of mice were recorded every three days, and the growth performance indices were calculated. No mortality was observed. The animals were not subjected to any painful procedures that required anesthesia and/or analgesia. All operators received training for the animal experiments as well as professional training in animal welfare ethics and were proficient in the relevant operating skills of this experiment.

**Table 2:** Animal group distributions and treatments.

Group		Intraperitoneal injection of reagents	Diet
CON	Acclimated for three days	Normal saline, once	Basal diet
CTX		Cyclophosphamide at 50 mg/kg BW, once	Basal diet
CTX + DON		Cyclophosphamide at 50 mg/kg BW, once	Basal diet with 12 mg/kg DON
CTX + DON + UGTs		Cyclophosphamide at 50 mg/kg BW, once	Basal diet with 12 mg/kg DON and 1 mg/kg UGTs

### Sample collection

At the end of the experiment, the mice were anesthetized with isoflurane, bled retro-orbitally to collect blood samples, and then euthanized by cervical dislocation to ensure death. Blood samples were separated by centrifugation, stored at − 80 °C for subsequent analysis, and the liver was stored at − 80 °C. A small part of the duodenum was soaked in 4% paraformaldehyde solution for histopathological observation, and the remainder was cut longitudinally after the fat on the surface was removed. The feces were rinsed with precooled physiological saline and stored at − 80 °C.

### Analysis of SOD, GSH, and MDA in the liver

The total glutathione (GSH) detection kit, total SOD activity detection kit (WST-8 method), lipid peroxidation MDA assay kit, and BCA protein concentration determination kit were purchased from Beyotime Institute of Biotechnology (Jiangsu, China) to detect the level of oxidative stress and the protein concentration of liver tissue in each group of mice. These results were read with a Spectra Max ABS plus full-wavelength enzyme labeling instrument (Molecular Devices, Shanghai, China).

### D-lactic acid and IgG analysis in serum

Serum D-lactic acid levels were detected using a D-lactic acid colorimetric test kit (Elabscience, Wuhan, China), and serum IgG levels were detected using a Mouse Immunoglobulin G (IgG) ELISA Kit (Sangon Biotech, Shanghai, China). The results were obtained with a SpectraMax ABS plus full-wavelength enzyme labeling instrument (Molecular Devices, Shanghai, China).

### Histological analysis

After the duodenum was fixed with 4% paraformaldehyde solution for 24 h, it was removed, embedded in paraffin wax and sectioned at a thickness of 5 μm. The sections were stained with hematoxylin and eosin (H&E) for histopathological analysis. Tissue morphology was examined with a light microscope (Model DP12, Olympus, Tokyo, Japan). The villus length and crypt depth of the duodenum were measured via ImageJ 1.8.0. The villus/crypt ratio was subsequently calculated.

### Extraction of mRNA and detection of relative gene expression levels

The total RNA of the duodenum was extracted with TRIzol reagent (Thermo Scientific, USA), the concentration of which was detected with a miniature ultraviolet spectrophotometer (NanoDrop 2000, Thermo Fisher Scientific, USA). cDNA was synthesized using Takara PrimeScript RT Master Mix (Takara Biomedical Technology Co., Ltd., Beijing, China) according to the manufacturer’s instructions. Relative gene expression levels were detected by Q-PCR technology, which was performed with Taq Pro Universal SYBR qPCR Master Mix (Vazyme, Nanjing, China) on a qTOWER^3^ Real-Time PCR System (Analytikjena, Germany), and the procedure was set as follows: preincubation at 95 °C for 30 s, 40 cycles of 95 °C for 10 s, 60 °C for 30 s. GAPDH was used to normalize mRNA expression levels. The relative abundance of mRNAs was calculated using the 2^−ΔΔct^ method. The sequences of the primers used were acquired from references or designed with Primer Premier 5.0 (Premier Biosoft, CA) and synthesized by Sangon Biotech; the primer information is shown in Table S1.

### Statistical analysis

All the data are presented as the means ± SEMs. The data were analyzed using SPSS (PASW Statistics 25.0, SPSS Inc., Chicago, IL, USA) software. Statistical analyses were performed using one-way ANOVA followed by the least significant difference (LSD) test, and bar charts were drawn via GraphPad Prism 8.0 (GraphPad Software, Inc., San Diego, CA) software. Differences among groups were considered statistically significant at *p* values < 0.05. Within the same indicator, different letters indicate significant differences (*p* < 0.05), whereas the same letter indicates insignificant differences (*p* > 0.05).

## Electronic supplementary material

Below is the link to the electronic supplementary material.

Supplementary Material 1

Supplementary Material 2

## Data Availability

All data supporting the findings of this study are available within the paper and its Supplementary Information.

## Abbreviations

UGTs: UDP-glycosyltransferases
DON: Deoxynivalenol
CTX: Cyclophosphamide
i.p.: Intraperitoneal injection
T-SOD: Total superoxide dismutase
GSH: Glutathione
MDA: Malonic dialdehyde
IgG: Immunoglobulin G
H&E: Hematoxylin and eosin
qRT-PCR: Quantitative real-time PCR
IFN-γ: Interferon-γ
IL-10: Interleukin-10
IL-13: Interleukin-13
Bax: Bcl-2-associated X protein
Bcl-2: B-cell lymphoma gene 2
P53: Transformation related protein 53
