# Supplementary material for: UDP-glycosyltransferases alleviate the toxic effects of deoxynivalenol on the growth performance and gut damage of Kunming mice
Source: Sci Rep. 2025 May 23;15:17989. doi: 10.1038/s41598-025-02712-6 (PMC12102272; doi:10.1038/s41598-025-02712-6)
Supplement: Supplementary file 1 — Supplementary Material 1 [file 41598_2025_2712_MOESM1_ESM.docx]

Supplementary materials

UDP-glycosyltransferases alleviate the toxic effects of deoxynivalenol on the growth performance and gut damage of Kunming mice

Jiaxu Liu ^1, ¶^, Xue Ling ^1, ¶^, Zhaoquan Chen^1^, Huajie Yang^1^, Sitao Guo^1^, Bingyang Zhou^1^, Pengwei Zhu^1^, Zheng Yang^1^, Yongqiang Wang ^1,*^

1 Key Laboratory of Microecological Resources and Utilization in Breeding Industry, Ministry of Agriculture and Rural Affairs, Guangdong HAID Group Co., Ltd, Guangzhou, China;

* Corresponding author.

E-mail address: wangyq04@haid.com.cn;

¶ These authors contributed equally to this work.

Table S1 Primer information.

| Genes | F/R | Primer sequence 5’→3’ | Accession number | Length | reference |
| --- | --- | --- | --- | --- | --- |
| IFN-γ | F | GTGAACAACCCACAGATCCAGC | NM_008337.4 | 101 bp | [1] |
|  | R | CGACTCCTTTTCCGCTTCCTTAG |  |  |  |
| IL-10 | F | GCTCTTACTGACTGGCATGAG | NM_010548.2 | 105 bp | [2] |
|  | R | CGCAGCTCTAGGAGCATGTG |  |  |  |
| IL-13 | F | CCTGGCTCTTGCTTGCCTT | NM_008355.3 | 116 bp | [2] |
|  | R | GGTCTTGTGTGATGTTGCTCA |  |  |  |
| Caspase-3 | F | TGGAGGCTGACTTCCTGTATGC | NM_001284409.1 | 174 bp | [3] |
|  | R | ATTCCGTTGCCACCTTCCTGTT |  |  |  |
| Bcl-2 | F | GGGATGCCTTTGTGGAACTATA | NM_009741.5 | 172 bp | [4] |
|  | R | CTTTTGCATATTTGTTTGGGGC |  |  |  |
| Bax | F | AGACACCTGAGCTGACCTTGGA | NM_007527.4 | 196 bp | [5] |
|  | R | TTGAAGTTGCCATCAGCAAACA |  |  |  |
| P53 | F | CCCCTGTCATCTTTTGTCCCT | NM_001127233.1 | 137 bp | [6] |
|  | R | AGCTGGCAGAATAGCTTATTGAG |  |  |  |
| Occludin | F | CTTTGGCTACGGAGGTGGCTAT | NM_001360538.1 | 86 bp | Premier 5.0 |
|  | R | CTTTGGCTGCTCTTGGGTCTG |  |  |  |
| Claudin1 | F | TCTACGAGGGACTGTGGATG | NM_016674.4 | 84 bp | [7] |
|  | R | TCAGATTCAGCAAGGAGTCG |  |  |  |
| Claudin5 | F | GCAAGGTGTATGAATCTGTGCT | NM_013805.4 | 109 bp | [8] |
|  | R | GTCAAGGTAACAAAGAGTGCCA |  |  |  |
| GAPDH | F | GGAGAAACCTGCCAAGTATG | NM_001411843.1 | 124 bp | [9] |
|  | R | GGAGTTGCTGTTGAAGTCG |  |  |  |

**Reference**

1. Liangxin, W., *Protective effects of aloe polysaccharides and aloin on aflatoxin B1-induced spleen injury in rats.* 2015, Jiangnan University: Wuxi, China.

2. Lan, T.J., et al., *Hydrolyzed seawater pearl tablet modulates the immunity via attenuating Th1/Th2 imbalance in an immunosuppressed mouse model.* J Tradit Chin Med, 2021. **41**(3): p. 397-405. <https://dx.doi.org/10.19852/j.cnki.jtcm.20210319.001>.

3. Yi, J., et al., *Battery wastewater induces nephrotoxicity via disordering the mitochondrial dynamics.* Chemosphere, 2022. **303**(Pt 1): p. 135018. <https://dx.doi.org/10.1016/j.chemosphere.2022.135018>.

4. Riyi, T., *Protective Effect and Mechanism of Taurine on AFB1 Induced Injury in Rats*. 2019, Shenyang Agricultural University: Shenyang, China.

5. Zhijie, D., *Protective effect of PBC2 on acute liver injury induced by AFB1 in rats*. 2018, Guangxi Medical University: Nanning, China.

6. Zhao, Y., et al., *He's Yangchao Recipe Ameliorates Ovarian Oxidative Stress of Aging Mice under Consecutive Superovulation Involving JNK- And P53-Related Mechanism.* Evid Based Complement Alternat Med, 2022. **2022**: p. 7705194. <https://dx.doi.org/10.1155/2022/7705194>.

7. Holmes, J.L., et al., *Claudin profiling in the mouse during postnatal intestinal development and along the gastrointestinal tract reveals complex expression patterns.* Gene Expression Patterns, 2006. **6**(6): p. 581-588. <https://dx.doi.org/10.1016/j.modgep.2005.12.001>.

8. Zhan, L., et al., *Toll-like receptor 4 deficiency alleviates lipopolysaccharide-induced intestinal barrier dysfunction.* Biomed Pharmacother, 2022. **155**: p. 113778. <https://dx.doi.org/10.1016/j.biopha.2022.113778>.

9. Jiaxu, L., et al., *Study on Detoxification Effect of Vomitoxin Oxidase.* Feed Review, 2022(004): p. 8-13. <https://dx.doi.org/10.20041/j.cnki.slbl.2022.04.002>.
